# Temporo-insular enhancement of EEG low and high frequencies in patients with chronic tinnitus. QEEG study of chronic tinnitus patients

**DOI:** 10.1186/1471-2202-11-40

**Published:** 2010-03-24

**Authors:** Morteza Moazami-Goudarzi, Lars Michels, Nathan Weisz, Daniel Jeanmonod

**Affiliations:** 1 Institute of Neuroinformatics, ETHZ/UNIZH, Winterthurerstrasse 190, 8057 Zurich, Switzerland; 2 University Hospital Zurich, Laboratory for Functional Neurosurgery, CH-8091 Zurich, Switzerland; 3 Department of Psychology, University of Konstanz, D-78464 Konstanz, Germany; 4 Center for Integrative Human Physiology, University of Zurich, CH-0857 Zurich, Switzerland

## Abstract

**Background:**

The physiopathological mechanism underlying the tinnitus phenomenon is still the subject of an ongoing debate. Since oscillatory EEG activity is increasingly recognized as a fundamental hallmark of cortical integrative functions, this study investigates deviations from the norm of different resting EEG parameters in patients suffering from chronic tinnitus.

**Results:**

Spectral parameters of resting EEG of male tinnitus patients (n = 8, mean age 54 years) were compared to those of age-matched healthy males (n = 15, mean age 58.8 years). On average, the patient group exhibited higher spectral power over the frequency range of 2-100 Hz. Using LORETA source analysis, the generators of delta, theta, alpha and beta power increases were localized dominantly to left auditory (Brodmann Areas (BA) 41,42, 22), temporo-parietal, insular posterior, cingulate anterior and parahippocampal cortical areas.

**Conclusions:**

Tinnitus patients show a deviation from the norm of different resting EEG parameters, characterized by an overproduction of resting state delta, theta and beta brain activities, providing further support for the microphysiological and magnetoencephalographic evidence pointing to a thalamocortical dysrhythmic process at the source of tinnitus. These results also provide further confirmation that reciprocal involvements of both auditory and associative/paralimbic areas are essential in the generation of tinnitus.

## Background

Tinnitus is an auditory phantom perception, reported subjectively as a tone and/or a noise, in the absence of an external stimulus [1]. Approximately 5-15% of the general population experience tinnitus [2]. In 1-3% of the general population the tinnitus affects the quality of life, involving sleep disturbance, work impairment and psychological distress [3,4]. The underlying physiological mechanisms that lead to phantom sensation are still being explored. In most cases, tinnitus is accompanied by an audiometrically measurable hearing loss, and even in a majority of those cases with normal audiograms abnormal outer or inner hair-cell function has been reported correlating with the presence of tinnitus [5,6].

Contemporary views of tinnitus emphasize the role of the central auditory system [1,4,7-14]. Studies in anaesthetized animals suggest enhanced firing rate and/or synchronized firing to be a necessary neurophysiological mechanism underlying tinnitus [9,15]. A reduction of tinnitus intensity in patients has been correlated to reduction of delta band power [16].

Alterations in spontaneous central neuronal activity patterns after peripheral deafferentations have recently been proposed to be essential in the genesis of tinnitus [6,17,18]. A relevance for peripheral deafferentation has also been proposed in the field of neurogenic pain, which prompted some authors to envisage that a similar mechanism might be at the source of tinnitus and neurogenic pain [19-23]. Peripheral deafferentation leads to thalamic desactivation [24], which in turn disrupts normal thalamocortical (TC) interaction [7,10], thus leading to the appearance of tinnitus. The effects of an abnormal thalamocortical interaction can be analysed at the cortical level using magnetoencephalogram (MEG) or electroencephalogram (EEG) [7,25]. This sequential view integrates both the induction in the periphery and the generation at the TC level of tinnitus. In the following, we refer to a mechanism that focuses on TC interplay [7]. First evidence for this mechanism in tinnitus was the finding of low-threshold calcium spike (LTS) bursts in the medial thalamus [10]. 50% of neuronal activity in the medial thalamus (central lateral nucleus, CL) was characterized as LTS bursts. LTS bursts displayed a delta/theta rhythmicity, with a mean interburst discharge rate of 4 Hz. LTSs have been described intracellularly in *in vitro *and *in vivo *experiments and have been related to a state of membrane hyperpolarization [26-28]. In tinnitus this would be a consequence of auditory deprivation caused by peripheral damage.

CL is part of the medial thalamus, is diffusely connected to wide cortical areas [29,30] and is thought to serve as a non-specific amplifier of TC activity [31]. The TC loop constitutes an important component contributing to the rhythmicity of scalp EEG and MEG [7,32]. The 4 Hz discharge rate of thalamic LTS bursts may be supposed to attract the TC system into low EEG frequencies, a proposition which is at the base of this and other studies. Thus analyzing spectral features of continuously recorded EEG offers a window for the investigation of abnormal TC interplay in tinnitus patients. Surprisingly, even though spontaneous activity has been a frequent research target in animal models of tinnitus, studies in humans have been rare. A few studies have looked at the resting oscillatory EEG/MEG in tinnitus patients as compared to healthy controls [7,25,33-37]. In a resting EEG study of patients with severe tinnitus as compared to healthy controls a significant increase of Z- score power over the frequency ranges from 0.5 to 22 Hz was reported [37], which was dominant in fronto-temporal electrodes. Another EEG study described an increase and decrease of average total power in female and male patients as compared to healthy controls, respectively [38]. A further study of spontaneous brain activity reported temporal and fronto-temporal changes (increases and decreases) of relative power in individuals with severe tinnitus [36]. Most recently, an MEG study found an increase and decrease of delta and alpha power respectively in tinnitus patients as compared to healthy controls [25]. Further studies emphasized the relevance of gamma (> 40 Hz) activity to the pathophysiology of tinnitus [34,35]. The aforementioned findings have been incongruous, showing both increase and decrease in different frequency band power. Therefore the characteristic of different frequency bands remains an ongoing debate in the pathogenesis of tinnitus. Otherwise, attempts to study tinnitus in humans have focused on the use of designs that measure neurophysiological responses to sounds [17,39,40] or experimental manipulations that enhance or reduce the perceived loudness [20,41-43]. In a recent experiment of that kind, Kahlbrock and Weisz [16] found transient reductions of tinnitus intensity following the offset of a masker (so-called residual inhibition, RI), accompanied by significant reduction in the delta frequency band. These changes were specific to a masker inducing RI and not observed with maskers that do not.

In light of the high variability of results we still lack sufficient knowledge of the anomalies of the resting EEG state in tinnitus patients, and the localization of cortical generators at the source of the observed power excesses has not been investigated in detail.

In the present study, we use power spectrum analysis and source localization of EEG data to identify cortical regions with changes in the underlying spontaneous activity patterns in individuals with tinnitus under both conditions eyes closed (EC) and eyes open (EO) as compared to healthy controls.

Our findings can be added to the microphysiological [10] and magnetoencephalographic [7,24,25] evidence for a thalamocortical dysrhythmic process at the source of tinnitus, characterised by a low frequency overproduction in thalamocortical loops.

## Methods

### Patients

The patient group consisted of 8 male patients (mean age 54 yrs, range 41-70 yrs). Patient demographics are summarized in Additional file 1: Table S1. Patients were referred by otorhinolaryngologists after all typical treatment options had been applied (see disease duration in Additional file 1: Table S1). The patient group was therefore characterized by chronic therapy resistant and outspoken tinnitus mechanisms.

### Healthy controls

The healthy control group consisted of 15 male subjects (mean age 58.8, 41-64 yrs). Statistical comparison revealed no statistical difference between the age of the healthy controls and the patient group (t-test, p = 0.36).

All control subjects were screened for health problems using a detailed health questionnaire (Zürcher Gesundheits-Fragebogen; Kuny and Stassen, 2004). They had no current or previous history of relevant physical illness and no current medication which could affect their EEG.

### EEG recording sessions

The study was approved by the Kanton Zurich ethics committee. All subjects, patients and controls were informed about the aim and the scope of the study and all gave written informed consent according to the declaration of Helsinki. Subjects were seated in a dimly lit room shielded against sound and stray electric fields and were video-monitored. All EEGs were acquired in the morning between 9 and 12 in order to exclude an impact of circadian factors on the EEG. Recording sessions of patients and controls followed an interleaved schedule and the recording apparatus was continuously calibrated. Subjects refrained from caffeinated beverages on the day of recording to avoid a caffeine-induced theta decrease in EEG [44]. Since drowsiness may result in enhanced theta power, the vigilance of subjects was checked by monitoring EEG parameters, such as slowing of the alpha rhythm or appearance of spindles. In addition, at the end of the recording, subjects were asked if they were awake during the whole recording session. Within each session, spontaneous EEG was recorded under two conditions: while subjects rested with their eyes closed, and while they rested with their eyes open. EEG was recorded for 5 min under each condition. Before each recording, subjects were instructed to assume a comfortable position in a chair. They placed their head on a chin-rest in order to minimize head-movements. For EC condition, subjects were instructed to place their fingers on their eyelids to reduce rolling eye movements and to relax but to stay awake. For eyes open condition, subjects were requested to keep eyes open and to maintain gaze on a fixation mark on the wall of the recording chamber. EEG signals were measured using 60 Ag/AgCl surface electrodes, which were fixed in a cap at the standard positions according to the extended 10-20 system (Easycap, Herrsching, Germany). During recording, electrode CPz served as reference. Impedances were below 5 kΩ in all electrodes processed in the further analysis. We used two additional bipolar electrode channels as eye and electromyogram (EMG) monitors. EEG signals were registered using the SynAmps EEG system (Neuroscan Compumedics, Houston, TX, 0.017 uV precision, sampling rate 250 Hz, 0.3-100 Hz analog band pass filter, -12 dB/octave) and continuously viewed on a PC monitor.

### Data preprocessing and editing

Data were analysed offline using Matlab (The Matworks, Natick, MA), EEGLAB (http://sccn.ucsd.edu/eeglab[45]), and custom scripts. The scalp EEG was re-referenced to the mean of the signals recorded at the ear lobes. Data were inspected in 5 s epochs, and artifacts from large muscle or eye movement were removed. For editing purposes, muscle artifacts were considered significant if the underlying EEG rhythms were not clearly seen. The EEG was decomposed into independent components using blind separation (independent component analysis, ICA). After the removal of components containing eye movement, muscle artifacts or heart beats, the signal was reconstructed. This procedure resulted in at least 143s of EEG per subject for estimates of power spectral density (mean: 278 ± 73s).

To investigate the effect of ICA component rejection, we compared tinnitus and control power spectra for EC condition in two approaches: 1) after visual artefact rejection only (before ICA) and 2) after additional ICA component rejection (after ICA). To test for significant differences between the two approaches we performed a repeated-measure ANOVA, considering mean band powers as within-subject variables and groups as between-subject variables. The mean power in the delta (2-4 Hz; F = 4.00, p = 0.06), theta (4-8 Hz; F = 2.00, p = 0.17), alpha (8-12 Hz; F = 0.75, p = 0.39), low beta (12-18 Hz; F = 1.55, p = 0.22), high beta (18-30 Hz; F = 1.86, p = 0.18), and gamma (30-100 Hz; F = 1.88, p = 0.18) frequency bands did not show a statistically significant difference between the two approaches. Therefore, we continue by reporting the results of ICA corrected data.

### Power spectral analysis

The spectral analysis was performed with the multi-taper method, which offers optimal spectral smoothing, and allows the trading of resolution in the frequency domain for reduced variance and has been described in details elsewhere (http://www.chronux.org[46]). Fourier transform was applied to tapered time series signal. We used an optimal family of orthogonal tapers (slepian functions). These are parameterized by their time length T and frequency bandwidth W. For chosen T and W, maximally k = 2TW-1 tapers well centred in frequency are appropriate for spectral estimation. Power spectra were estimated by time-bandwidth product TW = 4 and k = 3 tapers. The choice of the parameters depends on the available data set and could be made by iterative visual inspection [46]. In order to normalize the power spectra for each subject, the relative quantitative changes of power (ΔP) for each electrode were calculated by subtracting the average of the power spectra for each electrode of the subject for the EC condition.

### Statistical analysis of spectra

For groupwise comparisons, the power spectra of the tinnitus group and the healthy control group were compared with a non-parametric Wilcoxon rank sum test for each frequency point; Z-values were not corrected for multiple comparisons and were therefore considered as exploratory for frequency point comparisons.

For band power comparison, we applied a false discovery rate (FDR) [47] correction for multiple comparisons. The corrected significant p-values were verified to have at least p < 0.05.

### LORETA imaging

To localize the cortical sources of scalp EEG activity we used low resolution electromagnetic tomography analysis (LORETA; http://www.unizh.ch/keyinst[48]). The LORETA method is a discrete, three-dimensional (3D) distributed, linear, inverse solution. The smoothing constraints in LORETA endows the tomography with the property of low localization errors to test point sources, albeit with low spatial resolution (i.e. neighbouring neuronal sources will be highly correlated). The description of the method can be found in [48]. The LORETA inverse solution corresponds to the 3D distribution of electrical neuronal activity that features a maximum similarity (i.e. maximum synchronization), in term of orientation and strength, between neuronal populations in adjacent voxels. The imaging is therefore particularly tuned towards synchronized brain activities as they occur, e.g. in spreading oscillatory activations. Since LORETA takes explicitly into account that scalp electric potentials are determined up to an arbitrary additive constant, the final LORETA solution is independent of the electrical reference used. It is worth emphasizing that deep structures such as the anterior cingulate cortex [49], and mesial temporal lobes [50] can be correctly localized with LORETA. In the implementation of LORETA, computations were made in a head model using the MNI305 template [51], with the three-dimensional solution space restricted to cortical gray matter, as determined by the probabilistic Talairach atlas [52]. Voxel size is 7 mm on each side. Quantitative neuroanatomy (including Brodmann areas) were determined by the probabilistic Talairach atlas [52]. Anatomical labels such as Brodmann areas are also reported using MNI space, with correction to Talairach space [53]. In order to calculate tomographic LORETA images we first calculated the cross-spectral density matrix using multitaper FFT. With a spatial over-smoothing of 10^-4 ^the current source density was estimated for 2394 cortical voxels within the frequency bands given above [54]. This procedure resulted in one 3D LORETA image for each subject for each frequency range.

LORETA images were statistically compared between groups through multiple voxel-by- voxel comparisons in a nonparametric test for functional brain imaging [55]. The t-values corresponding to p < 0.01 or better were plotted onto a MRI template with a scale bar indicating statistical power and colour scale linearity equal to 75.

## Results

### Mean power spectra

**Figure 1:**
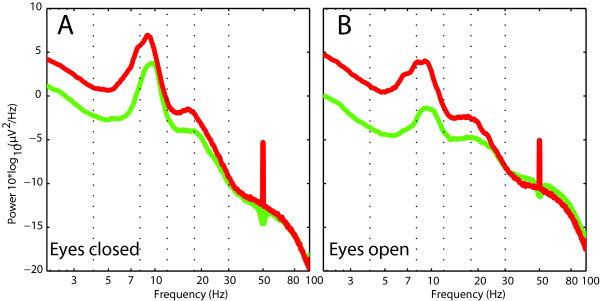
**Enhanced EEG power in tinnitus patients**. In global EEG power spectrum for the group of patients (red) was enhanced with respect to the group of healthy controls (green) in A and B for EC and EO conditions respectively.

In order to summarize the data and because spectra from all electrodes demonstrated similar shape and scale, we averaged the log-transformed spectra of all scalp electrodes for each subject. We then averaged these individual spectra to one spectrum for the patient group and one spectrum for the control group (Fig. 1A, B). The patient group exhibited more spectral power over the frequency range (2-100 Hz) for both the EO and EC conditions. The distribution of band power across the patient and control groups was significantly higher (p < 0.02) in the delta, theta and beta frequency bands, with slightly stronger effects for EO condition. Significant differences in delta, theta, and beta frequency bands (p < 0.02) were also demonstrated between patients and controls for the EC condition, both before and after normalization (see Additional file 2: Figure S1). Therefore, the increase in band power is unlikely to be due to a DC increase in power, which would be diminished or suppressed after normalization.

To investigate if there is any difference between patients with and without hyperacusis (see Additional file 1: Table S1), we performed a statistical unpaired two-sample t-test. There were no significant differences in the delta (p = 0.91), theta (p = 0.72), alpha (p = 0.60), beta1 (p = 0.88), beta2 (p = 0.92), and gamma (p = 0.72) spectral band power between the two subgroups.

### Scalp power topography

**Figure 2:**
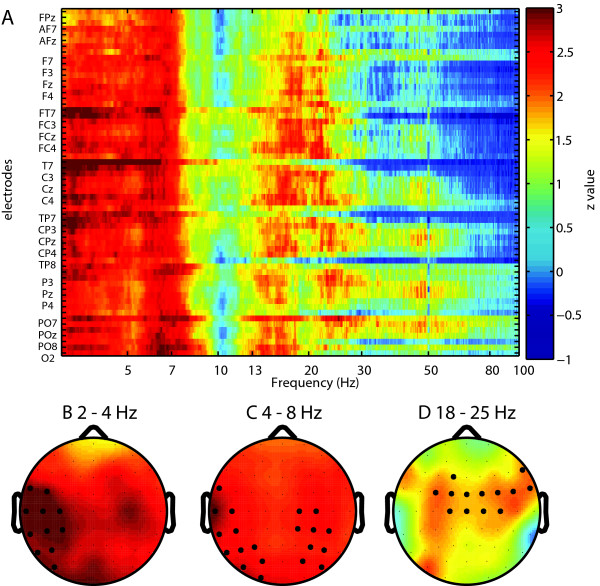
**Electrodewise comparison of power spectra for EC condition**. (A) Shown are Z-values for each electrode and frequency point (Wilcoxon rank sum tests). Non-corrected Z values above 1.96 correspond to p < 0.05. Highly significant electrodes were marked with black circles at different p values. (B) Delta (2-4 Hz) band (corrected, p < 0.004). (C) Theta (4-8 Hz) band (corrected, p < 0.016). (D) Beta (18-25 Hz) band (corrected, p < 0.05).

**Figure 3:**
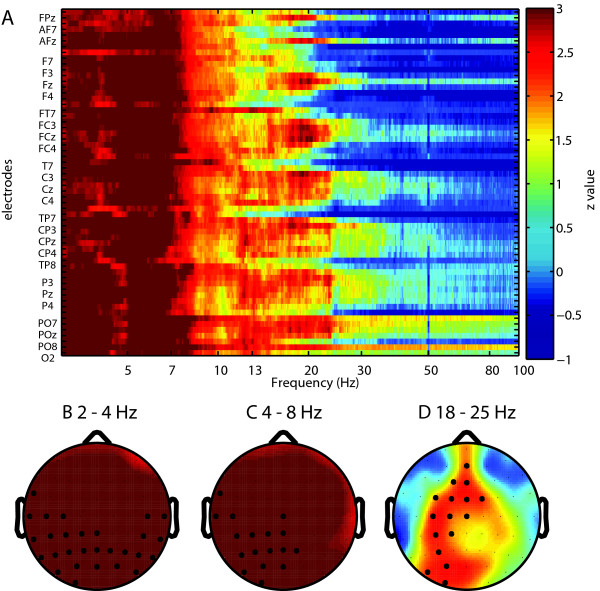
**Electrodewise comparison of power spectra for EO condition**. (A) Shown are Z-values for each electrode and frequency point (Wilcoxon rank sum tests). Non-corrected Z values above 1.96 correspond to p < 0.05. Highly significant electrodes were marked with black circles at different p values. (B) Delta (2-4 Hz) band (corrected, p < 0.0019). (C) Theta (4-8 Hz) band (corrected, p < 0.001). (D) Beta (18-25 Hz) band (corrected, p < 0.05).

After establishing a significant difference between patient and control group in spectra averaged over all electrodes, we were interested to know which electrodes contributed most to this difference and at which frequency. We performed Wilcoxon rank sum tests for each electrode at each frequency point and plotted the matrix of Z-values (Fig. 2A, Fig. 3A). Consequently we applied FDR correction for multiple comparisons for mean band power between the two groups, and significant electrodes were marked with black circles. At a higher statistical significance, delta increases were shown in left centro-temporo-parietal regions for the EC condition (corrected p < 0.004), and lateralized to the left more than right in the EO condition (corrected p < 0.0019). Theta increases were dominantly localized to the left temporo-parietal and right centro-parietal areas (corrected p < 0.016), in the EC condition, and left temporo-parieto-central regions (corrected p < 0.001) in the EO condition. The main beta increase is localized to left fronto-centro-parietal regions for the EC condition (corrected p < 0.05), and right fronto-central regions for the EO condition (corrected p < 0.05).

### LORETA source localization

**Figure 4:**
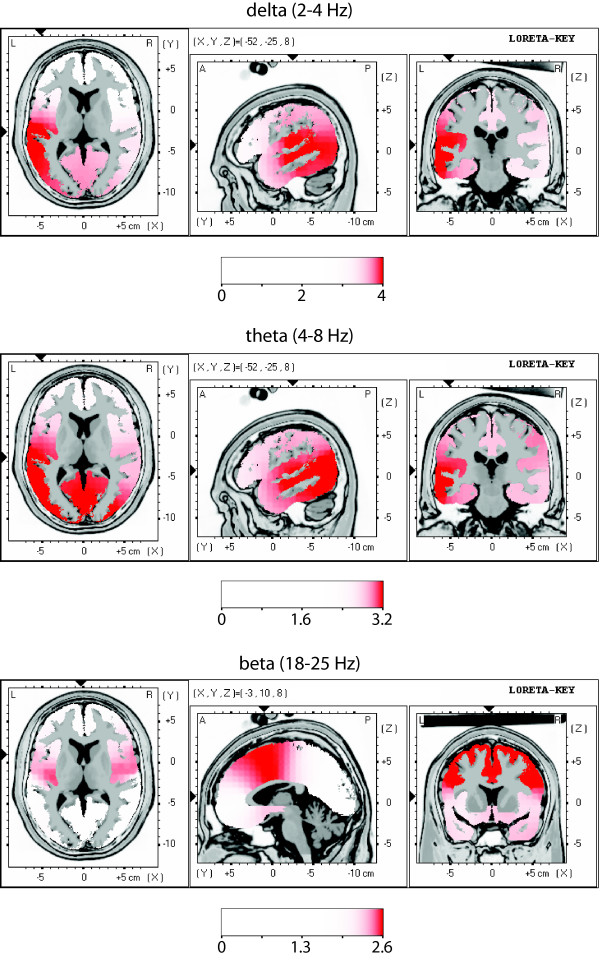
**Comparison of functional tomographic maps for EC condition**. LORETA functional images show differences in regional brain activity between patient group and healthy control group for three frequency ranges. For each frequency range, three orthogonal slices through the location of maximal increase are displayed. Images are colour coded (p < 0.05, corrected for multiple comparison; linearity = 75) registered to the stereotaxic Talairach space, and overlaid on a structural MRI scan. The MNI (Montreal Neurological Institute) coordinates are given on the slice maps. At cortical voxels, t values are colour coded according to the scale bar. Red areas correspond to overactivation in the patient group. Centre of overactivity for delta: BA 41, 42, 22, insula and 21; for theta: 41, 42, 22, insula, 23, 29, 30 and 21; for beta: insula, BA 24, 32, 6 and 9.

**Figure 5:**
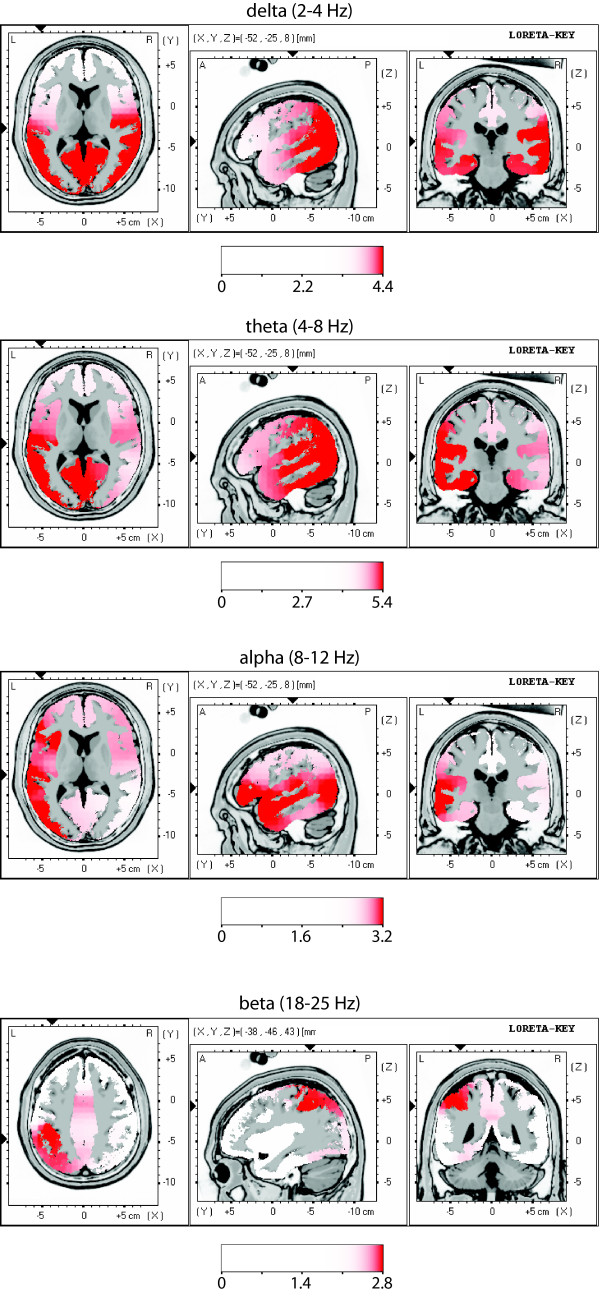
**Comparison of functional tomographic maps for EO condition for four frequency bands**. Center of overactivity for delta: BA 41, 42, 22, 23, 29, 30, insula, 21, parahippocampal gyrus; for theta: BA 41, 42, 22, 23, 29, 30, insula, 21, parahippocampal gyrus; for alpha: BA 41, 42, 22, insula, 21, prefrontal; for beta: BA 40.

Generators of differential power are shown as activation in statistical maps for different frequency ranges (Fig. 4, 5). The enhanced spectral EEG power of the patient group is reflected in cortical overactivation in delta, theta, alpha and beta frequency ranges focused over following cortical areas: auditory (BA 41, 42, 22), temporo-parietal (BA 21, 40), insular posterior, cingulate anterior (BA 24, 32), parahippocampal, and prefrontal/premotor (BA 6, 9). In the EC condition, the localization is left dominant (delta and theta) or bilateral (beta) (Fig. 4). In the EO condition, it is bilateral (delta) or left dominant (theta, alpha, and beta) (Fig. 5).

## Discussion and Conclusions

### Power

Our study on chronic tinnitus shows power enhancement of the spontaneous EEG activity, and localizes corresponding cortical generators of this dysrhythmic activity.

Our most striking finding in the EEG spectra of the patient group is the delta and theta EEG power enhancement (Fig. 1). An increase of cortical activity is in line with a previous MEG report of excess power in the whole frequency range for positive symptoms [7] and an increase of delta in tinnitus patients compared to healthy controls [25,36]. It is at variance with an earlier report [38]. This variance may be due to 1) the size of the patient groups and/or the selection of patients, and 2) the specific choice of frequency bands entering the statistical analysis. Our analysis is less likely to be biased in this respect, since Z-values are calculated for each frequency point. Concerning the statistics for band powers, we have corrected for multiple comparisons using FDR, yielding more potent statistical inference to our study.

The general increase in power in delta, theta and beta frequency ranges is confirmed if individual electrodes are analysed (Fig. 2A and Fig. 3A). This increase is generalized for theta and delta, and limited to fronto-centro-parietal sites in the beta domain. An increase of alpha is also found in the EO condition, localized on similar areas as delta and theta and indicating thus a participation of this frequency band in the thalamocortical dysrhythmia (TCD) tinnitus process. An increase in low and high frequencies is in line with the concept of TCD and may reflect the outspoken and chronic TCD involvement of our patient group [7,10,24,56]. This may explain the differences with the study of Weisz et al. [25], where only delta increase and a reduction of alpha were observed. Reduction of delta band power [16] and theta band power [57] after neurofeedback speaks for a causal relevance of slow oscillation increase in tinnitus. In an MEG case study, Llinas et al. [24] obtained a general (delta to beta) spectral power reduction after tinnitus masking.

### Cortical generators of excess EEG power

In the delta, theta, alpha and beta bands, the cortical generators of excess EEG power were located in dominantly left auditory (BA 41, 42, 22) temporo-parietal, insular posterior, cingulate anterior and parahippocampal cortical areas. Such a localization is in accordance with data from metabolic studies [58-63] and speaks for a dysrhythmic co-involvement of associative and paralimbic areas in the pathogenesis of tinnitus, which is consistent with their topographic and functional vicinity with the auditory system. Many other studies pointed out the involvement of associative/paralimbic areas [20,58,59,64] and the importance of reactive emotional factors in tinnitus has been repeatedly [1,17,34,61] reported, as well as reciprocal involvement of auditory and associative/paralimbic areas [12,61]. We observe a dominance on the left side for both TCD and tinnitus. At the time, we can only speculate that this observation is related to cognitive/emotional factors, e.g. the relevance of the non-acceptance of, and related frustration about, the presence of tinnitus. This non-acceptance may be viewed as primarily conceptual, i.e. left-side dominant. (see below the cognitive/emotional factor).

### Thalamocortical Dysrhythmia

**Figure 6:**
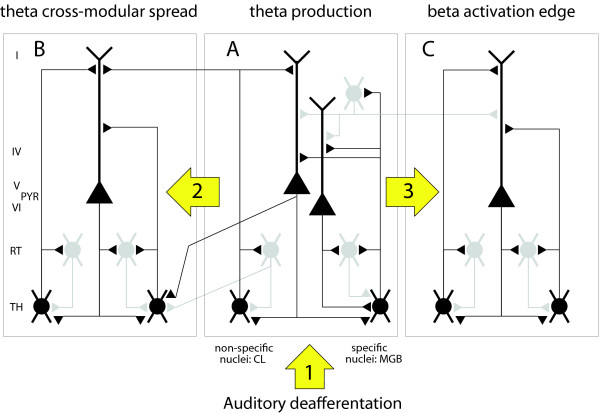
**Diagram of TC circuits**. Panels A, B and C show three TC modules which we consider to be identical [7]. Module A is depicted in more detail [30]. One module includes, first, cortical layers (I, IV, V and VI) with pyramidal cells and one grey GABAergic inhibitory cell; second, nucleus reticularis (RT, grey GABAergic inhibitory cells); and third, thalamus (TH), represented by one specific cell in medial geniculate body (MGB) and one non-specific cell (e.g. in CL). The specific thalamic cell projects to the apical dendrites of both layers V and VI pyramidal cells and collaterals sustain reticular feedback and cortical feed-forward inhibitions. The non-specific thalamic cell projects to RT and to the layer V pyramidal neuron and has a divergent connection onto the neighboring module. The corticothalamic feedback connection is depicted as intramodular onto RT and its thalamic relay cell, and divergent intra- and cross-modular onto 3 thalamic relay cells. There are also divergent cross-modular reticulothalamic projections. Arrows 1, 2 and 3 indicate the sequence of 1) auditory deafferentation, 2) theta cross-modular spread, and 3) beta activation edge.

Following the proposition of Llinas [7,24,65] of a central relevance of TC interaction in the genesis of hemispheric function and encouraged by our earlier finding of strong TC coupling [66,67], we propose here an interpretation of our results in the framework of TCD. This TC concept for neurogenic pain, abnormal movements, epilepsy, tinnitus and neuropsychiatric disorders was proposed [7,56,68] on the basis of experimental [26,28,29,69], and clinical evidence in the mentioned diseases [10]. It may be characterized by the following sequential set of events (schematized in Fig. 6):

(1) A lesion leads to deafferentation of excitatory inputs on thalamic relay cells and initiates the tinnitus syndrome (Fig. 6A). The deafferentation of excitatory inputs results in disfacilitation and cell membrane hyperpolarization.

(2) In the hyperpolarized state, deinactivation of calcium T-channels causes thalamic relay neurons to fire LTS bursts at delta/theta frequency [26].

(3) Bursting thalamic relay neurons exert a rhythmic influence on TC loops in the delta/theta frequency band. Thalamic and cortical areas are densely and reciprocally interconnected [29,30]. The tight functional coupling between thalamus and cortex is confirmed by the high theta coherence between the two [66,67]. This coupling is sustained by thalamocorticothalamic and also by thalamoreticulothalamic and corticoreticulothalamic recurrent projections [28]. The tendency of the TC network to maintain a given functional modality reinforces the hyperpolarized state over time [70].

(4) Divergent TC, corticothalamic and reticulothalamic projections provide the anatomical substrate for diffusion of low frequency activity to an increasing number of neighbouring TC loops (Fig. 6B: theta cross-modular spread).

(5) After recruitment of a sufficiently large number of TC loops, excess delta/theta power becomes measurable. Increased low-frequency oscillations also occur during sleep [28] and cognitive tasks [71,72], where they are considered as normal. It is the continuous, widespread and state-independent overproduction of slow rhythms in the awake brain that characterizes TCD.

(6) The final step towards the production of tinnitus is related to the reciprocal cortico-cortical inhibition mediated by GABAergic interneurons, which is a general feature of cortical organization (Fig. 6C). TC modules in delta/theta mode exert less collateral inhibition on neighboring modules, which are thereby overactivated in high (beta/gamma) frequencies. This event has been termed "edge effect" [7]. The concept is inspired by the effect of lateral inhibition in the retina. It has also been considered in the periphery of the auditory system [1]. The asymmetrical inhibition between a low frequency cortical area and neighbouring high frequency domains provides a ring of reduced inhibition onto, and thus activation of, the cortex surrounding this low frequency area. Support for such an effect was first provided by the increased interfrequency covariation between theta and beta ranges in MEG [7]. Recently, the increase of high frequency activation around a core of theta modules could be demonstrated in a slice preparation [24].

Plasticity mechanisms have been proposed to be at the base of the appearance of tinnitus. The following observations speak however for the necessity of at least another mechanism. The presence of a deficit after a damage to the auditory system and the induction of plastic mechanisms are both seen to happen in all deafferentation situations, whereas tinnitus, quite like neurogenic pain, develops and maintains itself along years only in a small (less than 10%) percentage of all deafferented subjects. Plastic map reorganizations, as argued by Weisz and collaborators [73], certainly contribute to the new post-lesional situation, but cannot satisfactorily explain the emergence and maintenance of tinnitus.

### The cognitive/emotional factor

Accumulating evidence from EEG and MEG studies underscores the fact that conceptual, mnestic [71,74,75] and emotional [76] activations increase hemispheric theta activity. Our EEG source localization data confirm other studies demonstrating the coactivation of cortical auditory and associative/paralimbic areas. This provides a substrate for a role of mental functions in the reactive modulation of neurogenic (i.e. due to auditory deafferentation) tinnitus and the generation of psychogenic tinnitus. Indeed, a top-down activation of the divergent corticothalamic projection from associative/paralimbic onto auditory areas, when strong and long-lasting enough to cause sustained thalamic overinhibition, might be at the source of an increase of theta production and, thus, of an amplification of neurogenic tinnitus mechanisms. It may even be at the origin of psychogenic tinnitus. One of the most deleterious conceptual/emotional dynamics is, in our experience, frustration and non-acceptance of the disease-related health impairment. The conceptual and practical consequences of these considerations are obvious and have a wide range of implications for the therapeutic support of neurogenic and psychogenic tinnitus patients. These include the recognition of a dual origination of tinnitus (neurogenic and psychogenic), in body and mind, respectively, but the existence of a common TC mechanism for both.

## List of abbreviations

BA: Brodmann area; CL: central lateral nucleus; EC: eyes closed; EO: eyes open; EEG: electroencephalogram; EMG: electromyogram; FDR: false discovery rate; FFT: fast Fourier transform; ICA: independent component analysis; LORETA: low resolution electromagnetic tomography analysis; LTS: low-threshold calcium spike; MEG: magnetoencephalogram; RI: residual inhibition; TC: thalamocortical; TCD: thalamocortical dysrhythmia.

## Authors' contributions

MMG conceived of the study, designed it, recorded the data, performed the data analysis, interpreted the data and drafted the manuscript. LM recorded the data and interpreted the data, NN recorded the data and interpreted of the data, DJ interpreted the data, drafted the manuscript and revised the manuscript critically. All authors read and approved the final manuscript.

## Supplementary Material

Additional file 1 **Table S1. Patient clinical details**. Symptom laterality: BL (bilateral), R (right), L (left). Quality of Life: QL. Intensity scaling for anxiety, depression, frustration, hyperacusis and reduction of QL: 0 is for normal, 1 for slight, 2 for moderate, 3 for strong and 4 for severe. VAS: visual analogue scale (0-100). NA: not available. Click here for file

Additional file 2 **Grand average of the normalized power spectrum for EC condition in tinnitus and healthy control groups**. Normalized EEG power spectrum for the group of patients (red) was enhanced with respect to the group of healthy controls (green). Click here for file
