# Supplementary material for: Temporo-insular enhancement of EEG low and high frequencies in patients with chronic tinnitus. QEEG study of chronic tinnitus patients
Source: BMC Neurosci. 2010 Mar 24;11:40. doi: 10.1186/1471-2202-11-40 (PMC2858736; doi:10.1186/1471-2202-11-40)
Supplement: Additional file 1 — Table S1. Patient clinical details. Symptom laterality: BL (bilateral), R (right), L (left). Quality of Life: QL. Intensity scaling for anxiety, depression, frustration, hyperacusis and reduction of QL: 0 is for normal, 1 for slight, 2 for moderate, 3 for strong and 4 for severe. VAS: visual analogue scale (0-100). NA: not available. [file 1471-2202-11-40-S1.PDF]

Table S1. Patient clinical details

Patient	Age (years)	Sex	Disease duration (years)	Side laterality (L,R,BL)	Tinnitus Localization	Tinnitus discription (Tone/Noise)	Mean tinnitus intensity (VAS 0-100)	Tinnitus definition by masking	Hyperacusis	Hypoacusis (subjective)	Hearing loss	Tinnitus Cause	Anxiety	Depression	Frustration	Reduction of QL
1	41	male	7	R	ear	noise	50	> 12 kHz	0	No	40 db at 8 kHz	emotional stress	0	2	2	3
2	34	male	12	BL	ears	tone/noise	42.5	NA	0	left 25% and Right 50%	NA	otosclerosis	1	1	2	2
3	50	male	2	R>L	ears and whole head	tone/noise	59	80 db	2	right 75%	subtotal	petrous bone fracture	2	1	2	3
4	63	male	20	L>R	ear and whole head	noise	77.5	85 db at 5500 Hz	0	left 25%	30 db at 500 Hz	auditory trauma and head trauma	2	2	2	3
5	46	male	14	L>R	ears and whole head	noise	100	NA	0	left 50%	15 db at 3 kHz	ENT operation and auditory trauma	2	1	3	3
6	64	male	2	BL	ears and whole head	tone/noise	45	45 db at 6 kHz	3	No	normal hearing	surgical stress	3	3	0	3
7	70	male	10	L	ear	noise	75	60 db at 8 kHz	2	left 25%	15 db at 8 kHz	depression	2	2	0	3
8	64	male	8	L	ear and left forehead	tone/noise	54	40 db at 1500 Hz	0	right 25% and left 100%	total deafness left side	Ménière and left VIII nerve neurectomy	0	1	3	3
median	56.5		9				56.5						2	1.5	2	3
mean	54.0		9.4				62.9						1.5	1.6	1.8	2.9
